# Cytokine Cocktail Promotes Alveolar Macrophage Reconstitution and Functional Maturation in a Murine Model of Haploidentical Bone Marrow Transplantation

**DOI:** 10.3389/fimmu.2021.719727

**Published:** 2021-09-21

**Authors:** Chao Hong, Hongyun Lu, Rong Jin, Xiaohong Huang, Ming Chen, Xiaoqiu Dai, Fangyuan Gong, Hongliang Dong, Hongmin Wang, Xiao-Ming Gao

**Affiliations:** Institutes of Biology and Medical Sciences, Soochow University, Suzhou, China

**Keywords:** bone marrow transplantation, immune reconstitution, alveolar macrophage, infection, cytokine

## Abstract

Infectious pneumonia is one of the most common complications after bone marrow transplantation (BMT), which is considered to be associated with poor reconstitution and functional maturation of alveolar macrophages (AMs) post-transplantation. Here, we present evidence showing that lack of IL-13-secreting group 2 innate lymphoid cells (ILC2s) in the lungs may underlay poor AM reconstitution in a mouse model of haploidentical BMT (haplo-BMT). Recombinant murine IL-13 was able to potentiate monocyte-derived AM differentiation *in vitro*. When intranasally administered, a cocktail of granulocyte-macrophage colony-stimulating factor (GM-CSF), IL-13, and CCL2 not only promoted donor monocyte-derived AM reconstitution in haplo-BMT-recipient mice but also enhanced the innate immunity of the recipient animals against pulmonary bacterial infection. These results provide a useful clue for a clinical strategy to prevent pulmonary bacterial infection at the early stage of recipients post-BMT.

## Introduction

Allogeneic hematopoietic stem cell transplantation (HSCT) is one of the most effective strategies to treat multiple diseases and disorders related with hematopoiesis failure, as well as blood malignancies. However, its success is greatly compromised by multiple post-transplantation complications, including graft *versus* host disease (GVHD), infection, and relapse of original disease, which may lead to transplantation failure and even patient death. Infectious complications are major cause of increased morbidity and mortality in HSCT recipients, which involve bacterial, viral, and fungal infections in different organs and tissues (1). Unfortunately, the development of effective treatment for infectious complications after HSCT is still challenging due to our poor understanding of the underlying immunological mechanisms. Infectious pneumonia is a common complication that occurs in a large group of HSCT recipients and remains a significant cause of mortality (2–4). Innate immune cells, especially neutrophils and macrophages, play critical roles in anti-pulmonary bacterial infection (5, 6). Whether donor stem cell rapidly and effectively engrafts and reconstitutes innate immune system is essential to protect HSCT recipients against challenging of respiratory pathogens. However, HSCT recipients beyond the period of neutropenia are also vulnerable to bacterial infection, which often sustain to the late post-engraftment period, indicating a long-term immune dysfunction of donor-derived innate immune system (7, 8).

Being recognized as the most important first-line pulmonary innate immune cells, alveolar macrophages (AMs) reside in alveoli and orchestrate both innate and adaptive immune responses to respiratory pathogens (9–11). Unlike classically activated inflammatory macrophages, residential AMs have high phagocytic capacity but are less efficient in triggering inflammatory immune response to respiratory bacteria (12–14). They patrol alveoli and phagocyte inhaled microbes, thus avoiding subclinical infection-induced neutrophil inflammation (15). Sufficient numbers of AMs are necessary to protect host against respiratory bacterial infection, as mice genetically deficient of AMs showed increased susceptibility to respiratory pathogens (16–18). Previous studies have shown that depletion of AMs through clodronate liposomes resulted in impaired host defense against pulmonary bacterial infection (15, 19). This is also evidenced by several studies focused on co-infection that influenza infection facilitates secondary bacterial infections by a mechanism of AM depletion (20–22). Developmentally, AMs are of embryonic origin and self-maintained throughout their lifespan independent of circulating monocyte differentiation and replenishment (23, 24). Under bone marrow (BM) transplantation (BMT) scenario, tissue-resident AMs can be eliminated and replaced by monocyte-derived AMs, which finally repopulate the alveolus space and functionally compensate the loss of host residential AMs (25–27). In syngeneic BMT models, it has been shown that AMs from late post-transplantation period display defective phagocytotic and killing ability, which impairs lung innate immune function against bacterial infection (28–30). Although PGE_2_ has been postulated as a master AM function modulator at post-engraftment period after BMT (29, 31), the underlying immunological mechanism of monocyte–AM differentiation still remains elusive. In clinical practice nowadays, haploidentical HSCT (haplo-HSCT) is more widely used due to the feasibility of selection of transplantation donors. However, infectious pulmonary complications still occur and remain as leading causes harmful for overall survival after haplo-HSCT (32, 33). According to the important role of AMs in anti-pulmonary infection, it is reasonable to assume that dysfunction of pulmonary innate immunity may be attributed to insufficient reconstitution and function of AMs after transplantation. However, detailed analysis of AM reconstitution after haplo-HSCT is still lacking. In this study, by using a mouse model of haplo-BMT, we demonstrate that lung group 2 innate lymphoid cell (ILC2)-derived IL-13 is critical for monocyte–AM differentiation after BMT and also that IL-13-based cytokine cocktail could effectively promote AM reconstitution post-BMT, thereby enhancing innate immunity against respiratory bacterial infection. The effect of GVHD on AM reconstitution after BMT was also assessed using this model.

## Results

### Delayed Alveolar Macrophage Reconstitution and Functional Maturation in B6D2F1 Mice Following Haploidentical Bone Marrow Transplantation

**Figure 1:**
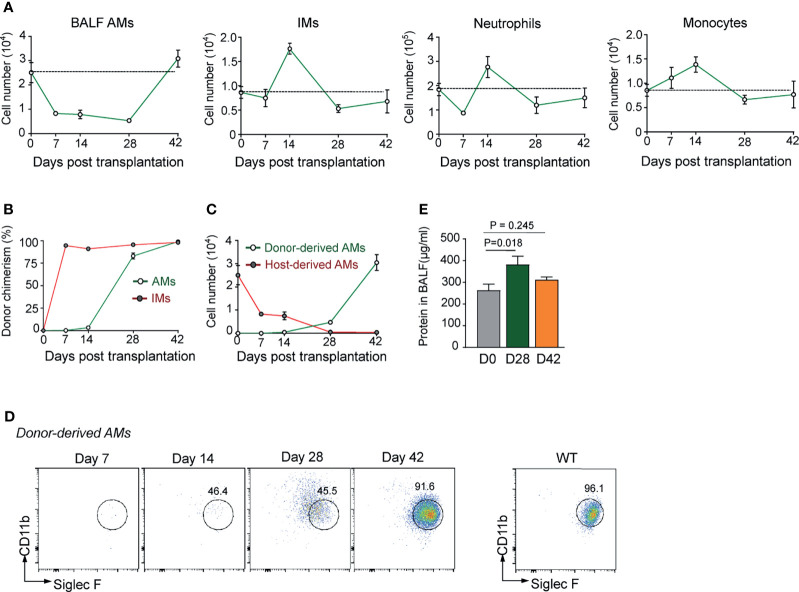
Defective reconstitution of AMs in allogeneic recipient mice after TCD-BMT. **(A)** The number of BAL AMs, lung tissue IMs, neutrophils, and monocytes was analyzed at different time points after BMT of allogeneic recipient mice. Data shown are the pooled results of two independent experiments with three mice per group in each experiment. **(B)** Donor chimerism of lung AMs and IMs in allogeneic recipient mice was analyzed by flow cytometry according to the cell surface expression of H-2D^b^ (donor) and H-2D^b/d^ (host). **(C)** The numbers of host-derived AMs and donor-derived AMs in allogeneic recipient mice were analyzed by flow cytometry. **(D)** The cell surface expression of CD11b and Siglec F was analyzed on donor-derived AMs at different time points in allogeneic recipient mice. AMs were firstly gated by expression of CD11c and F4/80. **(E)** Allogeneic recipient mice were sacrificed, and total protein concentrations in BAL fluid was measured by BCA Protein Assay kit. Total protein concentration in BAL fluid of WT mice was shown as control. Data shown are the pooled results of three independent experiments with three to four mice per group in each experiment. Data are represented as mean ± SEM. AM, alveolar macrophage; TCD, T cell-depleted; BMT, bone marrow transplantation; BAL, bronchoalveolar lavage; IM, interstitial macrophage; WT, wild type.

AMs are the most abundant immune cells residing in alveolus space, which represent over 95% cells in bronchoalveolar lavage (BAL) fluid of healthy mice. In the mouse model of haplo-BMT, T cell-depleted BM cells (TCD-BM) from healthy B6 mice were grafted into B6D2F1 recipients, followed by reconstitution and functional maturation analysis on AMs in BAL fluid at different time points post-transplantation. Gating strategy for flow cytometry analysis of AMs and other immune cells in BAL fluid and lung tissue is illustrated in **Supplementary Figure 1**. Interestingly, AMs freshly isolated from healthy B6D2F1 mice were able to proliferate in the presence of granulocyte-macrophage colony-stimulating factor (GM-CSF) (**Supplementary Figure 2**). By contrast, AMs from irradiated B6D2F1 mice were unable to do the same, indicating that AMs in irradiated recipient mice had lost self-renewal potential. As shown in **Figure 1A**, following allogeneic BMT, the total number of AMs in BAL fluid of recipient mice gradually dropped by approximately 70% from day 0 to day 14 (2.5 × 10^4^ *vs.* 0.8 × 10^4^/mouse) and remained as low until day 28. It was not until day 42 that the total number of AMs reached a normal level. In contrast, interstitial macrophages (IMs), neutrophils, and monocytes in lung tissue showed rapid reconstitution after BMT. Although total numbers of AMs recoverable from BAL fluid of recipient animals on day 14 and day 28 were similar, donor chimerism analysis showed a gradual replacement of host-derived AMs by donor-derived AMs from days 14 to 28 (**Figures 1B, C**). The time course of AM reconstitution is significantly slower than that of lung tissue-residing IMs. Donor-derived IMs repopulated lung tissue of recipient mice soon after BMT, constituting 90% of total IMs by day 7 (**Figure 1B**).

Although almost complete donor chimerism in AM population was observed by day 28 post-BMT, at this stage, donor-derived AMs still expressed CD11b at a high level (**Figure 1D**), a sign of monocyte origin rather than AM maturation. AMs are known to be responsible for maintaining alveolar microenvironment homeostasis by degrading lipids and proteins in alveoli (34, 35). Alveolar proteinosis in the recipient mice (**Figure 1E**) also provides circumstantial evidence for functional immaturity of the reconstituted AMs at this time point. These data confirm that, in irradiated BMT-recipient mice, empty niches formed by the loss of resident host AMs in the alveolus space were relatively slowly repopulated by AMs differentiated from donor monocytes, leaving a window of AM deficiency at early stage of BMT.

### Group 2 Innate Lymphoid Cells and IL-13 Deficiency in Lungs of Bone Marrow Transplantation Mice Correlates With Delayed Alveolar Macrophage Reconstitution

**Figure 2:**
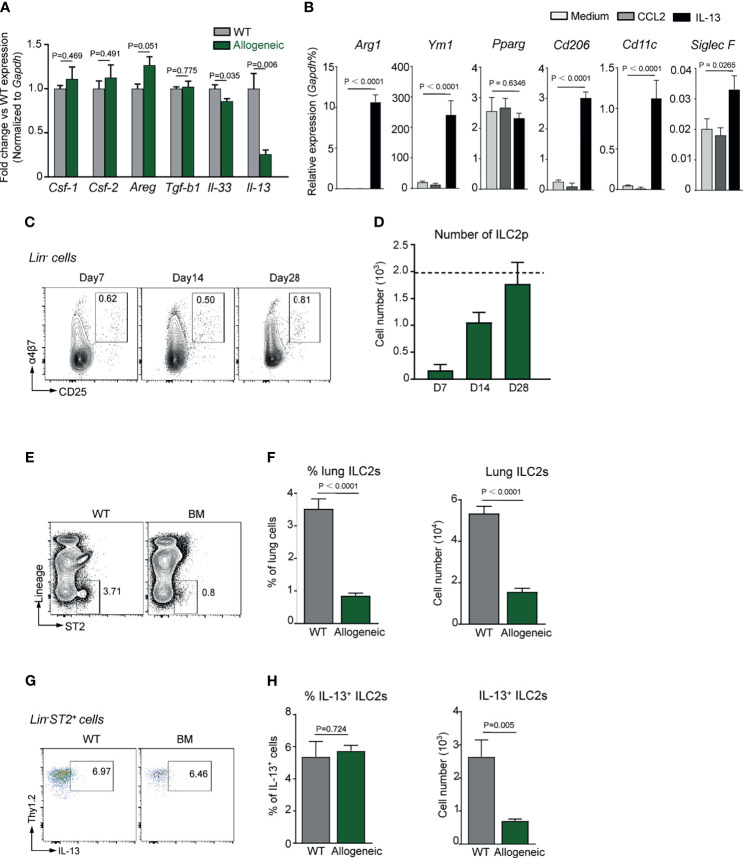
Defective ILC2s and IL-13 expression in allogeneic recipient mice after TCD-BMT. **(A)** Gene expression was analyzed in lungs of 28-day allogeneic recipient mice. Gene expression was normalized by relative fold change of each gene *versus* that in WT mice. A representative result of two independent experiments with four to five mice in each group is shown. **(B)** Monocytes were isolated from WT C57BL/6 bone marrow and cultured in the presence of 50 ng/ml of recombinant IL-13 or CCL2. Twenty-four hours later, gene expression was normalized to relative expression *vs. Gapdh*. A comprehensive analysis of three independent experiments is shown. Data are represented as mean ± SEM. **(C, D)** ILC2 precursors (Lin^−^α4β7^+^CD25^+^) were analyzed in bone marrow of allogeneic recipient mice after BMT. Dotted line indicates the level of ILC2p cells in WT mice. **(E)** Representative plots of lung ILC2 (Lin^−^ST2^+^) cells in lung of 28-day allogeneic recipient mice are shown. **(F)** Frequency and absolute number of lung ILC2s are shown. Data shown are the pooled results of two independent experiments with three to five mice per group in each experiment. Data are represented as mean ± SEM. **(G, H)** IL-13 production by lung ILC2s in mice were assessed by intracellular staining. Frequency and absolute number of lung IL-13^+^ ILC2s are shown. Data are represented as mean ± SEM (n = 5). ILC2, group 2 innate lymphoid cell; TCD, T cell-depleted; BMT, bone marrow transplantation; WT, wild type.

Cytokines such as GM-CSF, TGF-β1, IL-33, and IL-13 are known to be essential for AM differentiation and functional maturation *in vivo* (36–38). However, the transcription level of IL-13, but not M-CSF, GM-CSF, TGF-β1, and IL-33, in lung tissue of day 28 BMT mice was most significantly lower than that of un-transplanted control mice as evidenced by Q-PCR results (**Figure 2A**). It is thus reasonable to ask if deficiency in IL-13 expression in lungs was partially responsible for delayed AM reconstitution in TCD-BMT mice. To address this question, BM-derived monocytes were cultured in the presence of recombinant IL-13 or CCL2 for 24 h, followed by Q-PCR analysis on expression of AM signature genes. As illustrated in **Figure 2B**, in addition to enhanced expression of genes reminiscent of anti-inflammatory M2 macrophages (*Arg1*, *Ym1*, and *Cd206*), transcription of *Cd11c* and *Siglec5*, signature genes of AMs, significantly increased in cells stimulated with IL-13, but not CCL2.

It has previously been reported that, at steady state, IL-13 is exclusively produced by lung ILC2s (38). We therefore reasoned that the reduced IL-13 expression in lungs of BMT-recipient mice might be related to ILC2 deficiency. **Figures 2C, D** show that, on day 28 post-BMT, the number of ILC2 precursors (ILC2p) in recipient BM reached a normal level. However, the number of mature Lin^−^ST2^+^IL-13^+^ILC2 in recipient lung tissue was significantly lower than that of un-transplanted control mice (**Figures 2E–H**). It has previously been documented that lung basophils (Lin^−^CD11b^+^Fcεr1α^+^cKit^−^) regulate AM development and function *in vivo* (39), but no significant difference in basophil numbers was found between allogeneic recipient mice and the control mice (**Supplementary Figure 3**). These data endorse a likelihood that ILC2 deficiency and reduced IL-13 expression in the lungs of BMT mice are important factors underlying the poor AM reconstitution following TCD-BMT.

### Combination of IL-13, Granulocyte-Macrophage Colony-Stimulating Factor, and CCL2 Promotes Alveolar Macrophage Reconstitution in T Cell-Depleted Bone Marrow Transplantation Mice

**Figure 3:**
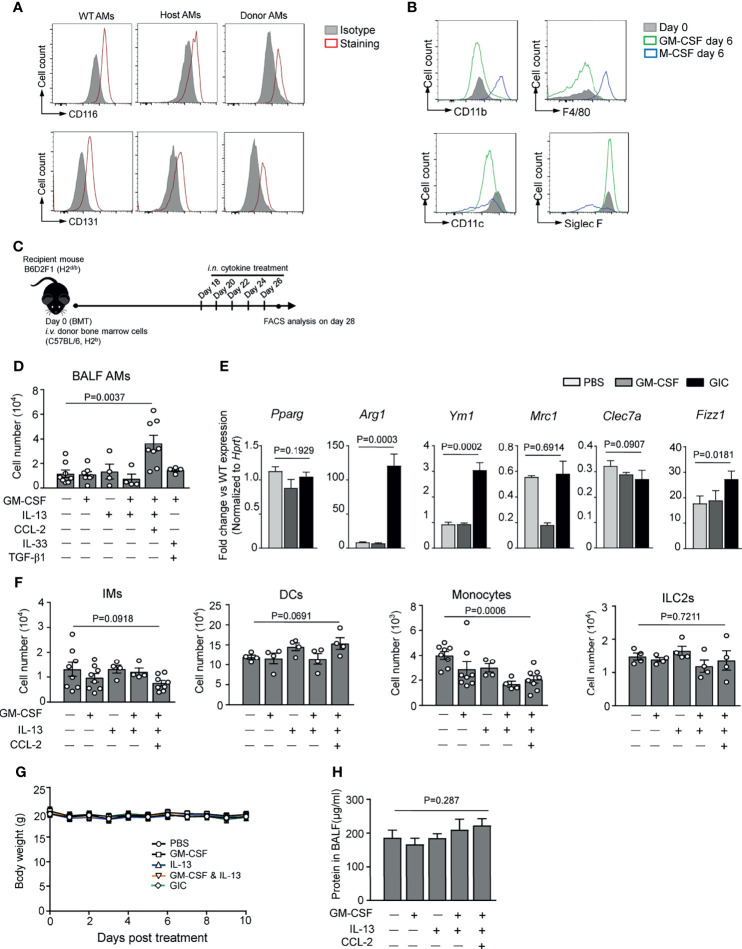
*In vivo* cytokine administration promotes AM reconstitution after TCD-BMT. **(A)** CD116 and CD131 expressions on host- and donor-derived AMs from day 14 and day 42 allogeneic recipient mice with TCD-BM grafts, respectively, were analyzed by flow cytometry analysis. BAL AMs from WT C57BL/6 mice were used as control. AMs from three mice in each group were equally pooled, and the expression of cell surface receptors was analyzed. **(B)** BAL AMs from WT C57BL/6 mice were cultured *in vitro* in the presence of 30 ng/ml of recombinant M-CSF or 30 ng/ml of recombinant GM-CSF for 6 days. Then cells were harvested, and cell surface expression of CD11b, F4/80, CD11c, and Siglec F was analyzed by flow cytometry. Freshly isolated WT AMs were used as control. **(C)** A schematic of i.n. cytokine treatment strategy after BMT. TCD-BMT-recipient mice were given different cytokines, alone or combination, started from day 18 every other day for a total of five times. **(D)** Two days after the last cytokine treatment, BAL AMs were analyzed in allogeneic recipient mice by flow cytometry. Data shown are the pooled results of two independent experiments with three to five mice per group in each experiment. **(E)** Q-PCR analyses of BAL AMs from allogeneic recipient mice treated with different cytokines *in vivo*. Gene expression was normalized by relative fold change of each gene *versus* that in AMs from WT mice. Data are represented as mean ± SEM (n = 3). **(F)** Lung tissue IMs, monocytes, DCs, and ILC2s were analyzed by flow cytometry in allogeneic recipient mice 2 days after the last time *in vivo* cytokine treatment. Data shown are pooled result of two independent experiments with three to five mice per group in each experiment. Data are represented as mean ± SEM. **(G)** Body weight of mice was monitored after the cytokine administration in different groups. **(H)** Two days after the last cytokine treatment, total protein concentration in BAL fluid of allogeneic recipient mice treated with or without cytokines was measured. Data are represented as mean ± SEM (n = 4). AM, alveolar macrophage; TCD, T cell-depleted; BMT, bone marrow transplantation; BAL, bronchoalveolar lavage; WT, wild type; IM, interstitial macrophage; DC, dendritic cell; ILC2, group 2 innate lymphoid cell.

The above results prompted us to explore the possibility of boosting AM reconstitution in BMT recipients by local administration of a cytokine cocktail with IL-13 as a major component, combined with GM-CSF, an essential differentiation/survival factor for myeloid cells (40), and/or CCL2, a monocyte chemokine. As shown in **Figures 3A, B**, host- and donor-derived AMs from BMT recipients strongly expressed CD116 and CD131 (GM-CSF receptors), and recombinant GM-CSF maintained cell surface expressions of CD11b, F4/80, and Siglec F by murine AMs *in vitro*. However, intranasally (i.n.) delivered IL-13 and GM-CSF, either alone or in combination, did not boost AM reconstitution in TCD-BMT mice (**Figures 3C, D**). On the other hand, cytokine cocktail containing IL-13, GM-CSF, and CCL2 (referred GIC below) exhibited a strong ability in promoting donor-derived AM expansion *in vivo*. IL-13 drives M2 polarization, which shares some common features with AMs (38, 41). M2 macrophages, defined by high-level expression of *Arg1*, *Fizz1*, *Mrc1*, and *Ym1*, play key roles in immune regulation and resolution of inflammation (42, 43). We next assessed the gene expression profile of AMs after GIC treatment. Q-PCR analyses showed that AMs from the GIC-treated TCD-BMT-recipient mice significantly elevated expression of M2-related genes, including *Arg1*, *Ym1*, and *Fizz1*, but not *Pparg* (**Figure 3E**). The difference made by CCL2 is likely due to its ability to recruit monocytes through CCR2 activation (44–46), rather than direct effect on AM expansion, since CCL2 alone was unable to stimulate AM differentiation *in vitro* (**Figure 2B**). GIC administration did not cause significant change in numbers of IMs, dendritic cells (DCs), monocytes, or ILC2 in lung tissue (**Figure 3F**) and was well tolerated by allogeneic TCD-BMT-recipient mice, as no body weight loss or protein concentration increase in BAL fluid was observed (**Figures 3G, H**). Collectively, GIC is effective and safe in promoting specific AM reconstitution in TCD-BMT recipients.

### Intranasal Administration of GIC Protects T Cell-Depleted Bone Marrow Transplantation Recipients Against Pulmonary Bacterial Infection

**Figure 4:**
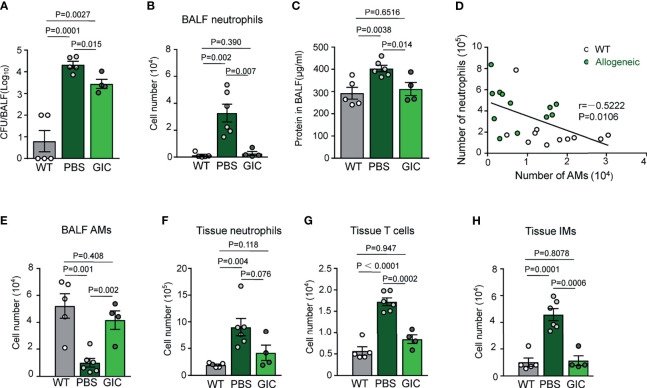
Promoting AM reconstitution increases the resistance of allogeneic recipient mice against respiratory bacterial infection after TCD-BMT. **(A)** Allogeneic recipient mice of TCD-BM grafts were i.n. treated with PBS or GIC started from day 16 every other day for a total of five times. Two days after the last treatment, these mice were infected with 2 × 10^5^ *Streptococcus pneumoniae via* i.n. 48 h later; bacterial CFUs were analyzed in BAL fluid. **(B)** Forty-eight hours after infection, neutrophils in BAL fluid were analyzed in infected mice by flow cytometry. **(C)** Total protein concentration in BAL fluid was analyzed in mice 48 h after *S. pneumoniae* infection. **(D)** WT mice and 28-day allogeneic recipient mice with TCD-BM grafts were infected with 2 × 10^5^ *S. pneumoniae via* i.n. Forty-eight hours later, the correlation between numbers of AMs and neutrophils in BAL fluid was analyzed. White circle, WT mice infected with *S. pneumoniae*; solid green circle, 28-day allogeneic recipient mice infected with *S. pneumoniae*. Data shown are the pooled results of three independent experiments with three to four mice per group in each experiment. **(E–H)** Forty-eight hours after infection, AMs in BAL fluid, neutrophils, T cells, and IMs in lung tissues were analyzed by flow cytometry. Data are represented as mean ± SEM with four to six mice in each group. AM, alveolar macrophage; TCD, T cell-depleted; BMT, bone marrow transplantation; PBS, phosphate-buffered saline; BAL, bronchoalveolar lavage; WT, wild type.

To address the question if GIC could boost innate immunity against pulmonary bacterial infection in TCD-BMT-recipients, a mouse model of *Streptococcus pneumoniae* infection, a leading cause of bacterial pneumonia in HSCT patients (4), was employed. In this model, mice were i.n. challenged with a sublethal dose of viable *S. pneumoniae* (2 × 10^5^ CFU/mouse), followed by numeration of bacteria and neutrophils in BAL fluid 48 h later. As illustrated in **Figures 4A, B**, healthy un-transplanted control mice almost completely cleared the infection 48 h after the challenge, as evidenced by very low bacterial burden and neutrophil infiltration in BAL fluid. By contrast, several magnitude higher bacteria burden was observed in BAL fluid of the BMT-recipient mice, indicating that innate immunity against bacterial infection was significantly weakened by the procedure of BMT. Bacterial growth in the alveolar space of the recipient mice was accompanied by infiltration of a large number of neutrophils, indicative of pneumonia. This model allowed us to test the effectiveness of GIC in enhancing innate immunity, *via* boosting AM reconstitution, against pulmonary bacterial infection in TCD-BMT recipients. Groups of mice were i.n. administered five doses of GIC, or phosphate-buffered saline (PBS) as control, from day 16 post-allogeneic TCD-BMT. Two days after the final treatment, the mice were i.n. challenged with a sublethal dose *S. pneumoniae*, followed by assessment of bacterial load and immune cell composition in BAL fluid and lung tissues. As presented in **Figures 4A–C**, bacterial burden, neutrophil infiltration, and proteinosis in BAL fluid of the GIC group were significantly lower than those of the PBS control group. The beneficial effects of inhibiting neutrophil infiltration during infection are most likely the result of AM reconstitution in TCD-BMT recipients, as evidenced by a clear negative correlation between neutrophil and AM numbers in these mice (**Figure 4D**). In the homogenized lung tissues, lower rather than higher numbers of neutrophils, T cells, and IMs were observed in the GIC group (**Figures 4E–H**). Collectively, GIC treatment is an effective way to reinforce the resistance of allogeneic recipient mice against pulmonary bacterial infection through promoting donor-derived AM reconstitution after TCD-BMT. The beneficial effects of GIC treatment in TCD-BMT recipients may include i) more effective clearance of pulmonary bacterial infection; ii) protection against infection-induced damage to epithelium lining the alveolar surface; and iii) better controlled airway inflammation.

### Role of Graft *Versus* Host Disease on Alveolar Macrophage Reconstitution and Anti-Infection Immunity

**Figure 5:**
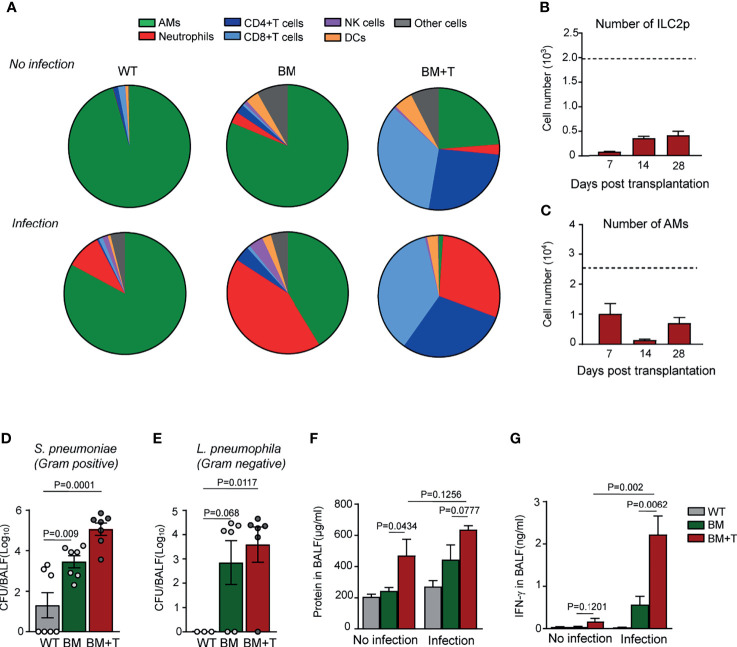
AM reconstitution and anti-infection immunity in mice with GVHD. **(A)** Ten days after BMT, allogeneic recipient mice were infected with 2 × 10^5^ *Streptococcus pneumoniae via* i.n. 48 h after infection, immune cells in BAL fluid were analyzed, and percentages of these cells were shown. Cells in mice without infection were also analyzed by flow cytometry. **(B)** ILC2 precursors (Lin^−^α4β7^+^CD25^+^) were analyzed in bone marrow of allogeneic recipient mice received both BM grafts and splenic T cells. Dotted line indicates the level of ILC2p cells in WT mice. Data are represented as mean ± SEM (n = 3). **(C)** The number of BAL AMs was analyzed at different time points of GVHD mice after BMT. Dotted line indicates the level of BAL AMs in WT mice. Data shown are the pooled results of two independent experiments with three mice per group in each experiment. **(D)** CFUs of bacteria in BAL fluid were analyzed 48 h after *S. pneumoniae* infection. Data are represented as mean ± SEM. **(E)** Ten days after BMT, allogeneic recipient mice were infected with 5 × 10^6^ *Legionella pneumophila via* i.n. 48 h after infection; CFUs of bacteria in BAL fluid were analyzed. WT mice infected with the same amount of bacteria were used as control. **(F)** Total protein concentrations and **(G)** IFN-γ in BAL fluid were analyzed. WT mice infected with the same amount of bacteria were used as control. Data shown are the pooled results of two independent experiments with three to four mice per group in each experiment. Data are represented as mean ± SEM. AM, alveolar macrophage; GVHD, graft *versus* host disease; BMT, bone marrow transplantation; BAL, bronchoalveolar lavage; ILC2, group 2 innate lymphoid cell; WT, wild type.

T cell-mediated GVHD is considered to play a harmful role in immune reconstitution after BMT, but the molecular mechanisms remain so far elusive. We addressed the question if GVHD would further delay AM reconstitution following BMT by using a GVHD mouse model, in which irradiated B6D2F1 mice were grafted with BM plus purified splenic T cells (BM+T) from donor mice. Ten days after BM+T transplantation, GVHD was evident by the large number of T lymphocytes in their BAL fluid of the recipients (**Figure 5A**), which was lethal within 42 days. Compared with slow recovery of ILC2p in TCD-BMT mice (**Figure 2D**), ILC2p numbers in the BM of BM+T recipient mice remained very low even 28 days post-transplantation (**Figure 5B**), mirrored by almost undetectable IL-13-producing ILC2 cells in the lung tissue (data not shown). This well coincides with complete loss of AMs in the lung tissues 14 days post-BM+T grafting, compared with their TCD-BMT counterparts (**Figures 1A**, **5C**). When allogeneic recipient mice of the BM+T and BM groups were compared in *S. pneumoniae* challenge experiments, approximately 40-fold higher bacterial burdens were found in BAL fluid of the BM+T group (**Figure 5D**), which was accompanied by overwhelming T-cell and neutrophil infiltration into alveolus spaces, as evidenced by flow cytometry analysis of cells in BAL fluid (**Figure 5A**). Similar results were observed when we use *Legionella pneumophila*, a causative pathogen of Legionnaires’ disease (47, 48), to infect allogeneic recipient mice (**Figure 5E**). Recipients of the BM+T grafts showed more severe epithelial damage than recipients of TCD-BM grafts alone, evidenced by significantly increased protein concentration in BAL fluid, but bacterial infection did not seem to cause drastic alteration in this aspect (**Figure 5F**). IFN-γ produced by T cells primes AMs during infection and promotes trained immunity of AMs against infection (49, 50). We found much higher levels of IFN-γ in BAL fluid of GVHD mice than non-GVHD mice after infection, indicating a strong T cell and/or NK cell response in lungs in these mice (**Figure 5G**). AMs from GVHD mice also expressed elevated levels of cell surface MHC class I after infection (**Supplementary Figures 4A, B**). However, BMT mice that received BM+T grafts failed to eliminate respiratory bacteria 48 h after infection (**Figure 5E**). Together, these results suggest that T cell-mediated GVHD could further delay AM reconstitution after BMT, complicate AM-dependent anti-bacterial defense, and exacerbate inflammatory responses after pulmonary bacterial infection.

## Discussion

**Figure 6:**
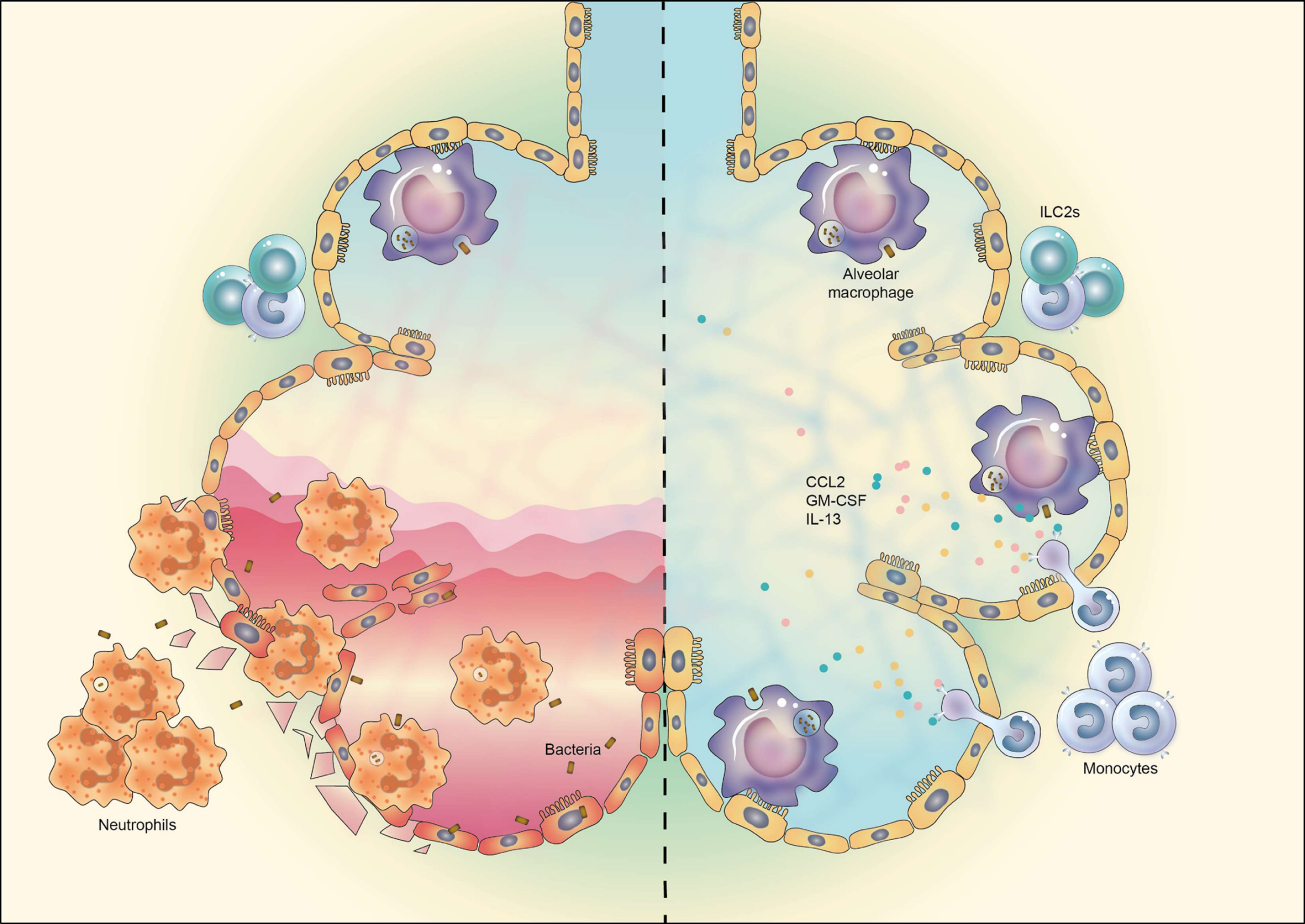
Schematic representation of manipulation of AM reconstitution *in vivo* to enhance the resistance against respiratory bacterial infection after TCD-BMT. The reconstitution of donor monocyte-derived AMs after TCD-BMT is defective due to the insufficient support of lung microenvironment, attributable mainly to reduced ILC2 cells and IL-13 production. TCD-BMT mice fail to eliminate inhaled pulmonary bacteria *via* AM-dependent immunity, which causes epithelia cell damage and severe lung inflammation dominated by neutrophil infiltration (*left*). Exogenous administration of a cytokine cocktail containing recombinant murine GM-CSF, IL-13, and CCL2 could promote donor monocyte-derived AM reconstitution and thus enhance the resistance of TCD-BMT mice against pulmonary bacterial infection (*right*). AM, alveolar macrophage; TCD, T cell-depleted; BMT, bone marrow transplantation; GM-CSF, granulocyte-macrophage colony-stimulating factor.

AMs are known to be the most important players in maintaining immune homeostasis in lung and fine-tuning immune responses to respiratory pathogens (51, 52). In haplo-BMT-recipient mice, however, AM reconstitution is very slow (AM numbers in BAL fluid of recipient mice 28 days after BMT were approximately 20% of that of untreated control mice), thereby resulting in increased susceptibility to pulmonary infection. A similar correlation between delayed AM reconstitution and susceptibility to pulmonary infection has been observed in HSCT patients (4, 8, 53). Therefore, strategies to boost AM differentiation post-BMT are of importance. Our data show that administration of recombinant IL-13, together with recombinant CCL2 (for monocyte recruitment) and GM-CSF (for monocyte and AM survival), could effectively promote donor-derived AM reconstitution *in vivo* and thus enhance the resistance of TCD-BMT recipients against respiratory bacterial infection (**Figure 6**).

One reason for the slow AM reconstitution in BMT recipients is that only donor-derived peripheral blood monocytes could serve as AM precursors; and their recruitment, survival, and differentiation require sufficient supply of vital cytokines such as CCL2, GM-CSF, and IL-13. Our study uncovers ILC2 deficiency and reduced IL-13 secretion thereof in the lungs as an important reason behind slow AM reconstitution. ILC2s are known to maintain M2 polarization and function of AMs *via* production of IL-13 (38). They constitute a dominant ILC population in lung and have been reported to promote the repair of lung epithelial cells to restore the epithelial integrity *via* production of amphiregulin (AREG) and thus increase the resistance of host to infection and decrease the pathogen burden in lungs (54). CCL2/CCR2 axis is known to be critical for monocyte recruitment (55). CCL2 administrated intratracheally into mice recruited monocyte migration into alveoli and lung tissue (46), but whether CCL2 could drive macrophage polarization remains so far controversial (56–61). On the other hand, compared with IL-13, CCL2 alone was not able to stimulate M2 differentiation *in vitro*. GM-CSF is the most important growth factor that could support AM development *in vivo* (34). It can also act as an essential survival factor for monocytes and macrophages both *in vitro* and *in vivo*. AMs, but not IMs, proliferate in the presence of GM-CSF (40). It seems that the effect of promoting AM reconstitution is dependent on specific cytokine combination, as another cytokine cocktail containing GM-CSF, IL-33, and TGF-β1 showed no significant impact on AM reconstitution after TCD-BMT (**Figure 3D**). The impact of GIC on AM reconstitution is most likely a combinational effect of cytokines. Monocytes attracted by CCL2 differentiate into M2-like AMs under the stimulation of IL-13, while GM-CSF could further support their survival and proliferation *in vivo*. However, it still remains to be determined whether other cytokine combinations are effective as GIC in promoting AM reconstitution *in vivo*. Considering the important role of GM-CSF in AM development and conflicting expression of *Csf-2* in day 28 in lungs of TCD-BMT mice (**Figure 2A**), future studies are required to understand the importance of exogenous supply of GM-CSF and the underlying mechanism on AM reconstitution *in vivo* after TCD-BMT. Systemic analysis of the dynamic change of cytokine expression profile in lung post-BMT will be of interest that may benefit the discovery of new factors and optimization of cytokine treatment approach in promoting AM reconstitution *in vivo*.

Domingo-Gonzalez and colleagues recently reported that AMs from 5-week syngeneic BMT mice exhibited differential phagocytic ability to *Pseudomonas aeruginosa* and *Staphylococcus aureus*, dependent on PGE_2_-mediated alterations in scavenger receptor and miR-155 expression (31). Interestingly, impaired killing ability of BMT AMs led to increased susceptibility to pulmonary bacterial infection (31). In addition to the dependency of their functional maturation, the capacity of AMs against pulmonary infection is limited by their cell number. Once the capacity of AMs is overwhelmed, respiratory pathogens as well as their derivative PAMPs stimulate lung epithelial cells to produce multiple cytokines and chemokines to induce the recruitment and activation of cells of lung immune system (62). Excessive inflammation leads to severe pneumonia and damage lung function (63). Mice that received BM+T grafts developed GVHD, which is the main reason for mortality around 4 weeks post-BMT (data not shown) and also renders these mice vulnerable to experiment procedures in the late period post-BMT. Nevertheless, it should be noted that GVHD is another reason for increased lung inflammation and impaired immune response against respiratory infection. GVHD complicated AM reconstitution and AM-dependent immune response against respiratory bacterial infection, which made it hard to evaluate the function of AMs in GVHD recipient mice. In this study, our data provided evidence that GVHD deteriorated delayed AM reconstitution after BMT and further impaired the ability of recipients against pulmonary bacterial infection. However, whether cytokine treatment is effective on AM reconstitution in GVHD recipients needs further investigation.

It has been reported that CD8 T cells prime AMs during infection *via* their production of IFN-γ, which is required for induction of trained immunity of AMs against infection (49). In our study, mice that received BM+T grafts showed significantly increased IFN-γ in BAL fluid, and AMs elevated their cell surface expression of MHC class I after infection (**Supplementary Figure 4**). However, IFN-γ priming did not enhance AM-dependent elimination of respiratory bacteria in mice with BM+T grafts (**Figures 5D, E**). Mouse models of BM+T grafts could not represent current clinical procedure of haplo-HSCT, a majority of which are performed as T cell-replete (TCR) haplo-HSCT following *in vivo* T-cell depletion or inhibition by using anti-thymocyte antibodies or cyclophosphamide in the early post-transplantation period (64–68). TCD-haplo-HSCT is often considered with higher incidence of infection and non-relapse mortality due to delayed immune reconstitution, compared with TCR-haplo-HSCT (69–71). Our findings in this study provide clear evidence that promoting AM reconstitution could protect mice against respiratory bacterial infection after TCD-BMT. Considering the broad use of TCR-haplo-HSCT in patients, AM reconstitution and function in TCR-BMT settings merit further investigation.

In summary, the results of this study demonstrated that delayed and defective reconstitution of AMs in allogeneic recipient mice acted as a vulnerability to pulmonary bacterial infection after TCD-BMT. Lack of supportive lung microenvironment, mainly attributed to deficient ILC2 and IL-13 production, impaired effective monocyte-derived AM reconstitution after TCD-BMT, which could be rescued by exogenous supply of cytokine cocktail containing recombinant IL-13, GM-CSF, and CCL2. Our data reveal the mechanism of AM reconstitution in a mouse haplo-TCD-BMT model and provide a foundation on the development of clinically applicable method to enhance the resistance of HSCT patients to pulmonary bacterial infection *via* promoting AM reconstitution.

## Materials and Methods

### Mice

B6D2F1 mice (H-2^b/d^) were bred at the Soochow University Animal Facility from female C57BL/6 (H-2^b^) and male DBA/2 (H-2^d^) mice or purchased from the Model Animal Research Center of Nanjing University (Nanjing, China) and used for experiments. C57BL/6 and DBA/2 mice were purchased from the Beijing Vital River Laboratory Animal Technology Company. Mice were kept in a specific pathogen-free facility in microisolator cages, and experiments were performed with mice at 8–10 weeks of age. All animal protocols were approved by the Institutional Laboratory Animal Care and Use Committee of Soochow University.

### Bone Marrow Plantation Models

BM cells from C57BL/6 mice were removed aseptically from femurs and tibias, and T cells were depleted by incubation with anti-Thy 1.2 antibody for 30 min at 4°C, followed by incubation with Low-TOX-M rabbit complement (Cedarlane) for 40 min at 37°C. Splenic T cells were purified by positive selection using anti-CD5 antibody-conjugated magnetic beads (Miltenyi, Auburn, CA). TCD-BM cells of 5 × 10^6^ with or without 1 × 10^6^ splenic T cells (to induce GVHD) were washed and resuspended in PBS and injected into lethally irradiated recipient B6D2F1 mice through the lateral tail vein. B6D2F1 mice received a total of 11-Gy total-body irradiation with a split of 3 h interval to minimize the gastrointestinal toxicity.

### Monocyte Isolation and Culture

Monocytes were isolated from wild-type (WT) C57BL/6 mice BM with EasySep™ mouse monocyte isolation kit (STEMCELL). After isolation, monocytes (above 90% purity) were cultured in Roswell Park Memorial Institute (RPMI) 1640 containing 10% fetal bovine serum (FBS) in the presence of 20 ng/ml of recombinant murine IL-13 or not, at 37°C, 5% CO_2_ in a humidified incubator. Twenty-four hours later, cells were collected for gene expression analysis.

### Analysis of Bronchoalveolar Lavage Fluid and Lungs

For BAL fluid collection, a small cut was made in the trachea, and a catheter was inserted. Lavage was performed three times with 800 μl of ice-cold PBS each time. Lavage fluid of first and second lavage was used for total protein and bacterial colony analyses. Fluid of three lavage was mixed together and centrifuged to pellet cells for flow cytometry analysis. Total protein was measured with BCA Protein Assay kit according to the manufacturer’s instructions (Pierce Thermo Scientific). After BAL, lungs were perfused with 10 ml of ice-cold PBS through the right ventricle of the heart. Lungs were dissected and cut into small pieces and then incubated for 1 h at 37°C with shaking in Hanks’ Balanced Salt Solution (HBSS) containing 10% FBS, 1 mg/ml of collagenase D (Sigma-Aldrich), and 25 μg/ml of DNAse I (Sigma-Aldrich). The lung homogenate was passed through a 70-μm cell strainer, and cells in flow-through were harvested, centrifuged, and treated with red blood cell lysis buffer (CWBIO, China) for 5 min. The cells were washed and resuspended in PBS containing 0.1% bovine serum albumin (BSA) and stained with fluorescence conjugated mAbs for flow cytometry analysis. In some cases, the left lobes of lungs from mice were homogenized in guanidinium thiocyanate (GTC) buffer (Omega) for total RNA extraction and followed by gene expression analysis.

### Quantitative Real-Time PCR

Total mRNA was isolated using the E.Z.N.A. HP Total RNA Kit according to the manufacturer’s instructions (Omega). cDNA was generated with SuperScript reverse transcriptase (Invitrogen). SYBR Green Master Mix (Applied Biosystems), commercially ordered primers, and an ABI7500 real-time PCR system (Applied Biosystems) were used for quantitative real-time PCR amplification of cDNA. Results were presented as relative expression normalized to those of *Gapdh* or *Hprt* expression or as fold changes *versus* indicated controls. Relative expression was calculated as “(2 ^(CT test − CT ref)^) × 100%.” Primer sequences are listed in **Table S1**.

### Bacterial Strains and Infection Experiment

For infection experiments, *L. pneumophila*, Philadelphia 1 (American Type Culture Collection (ATCC) 33152), was inoculated from a frozen stock onto buffered charcoal yeast extract (BCYE) agar plates at 37°C for 4 days. *S. pneumoniae* serotype 3 (ATCC 6303) was inoculated from a frozen stock onto sheep blood agar plates at 37°C overnight. After culture, bacteria were harvested by rinsing plates with PBS, pelleted by centrifugation, and resuspended in PBS (CFUs estimated by OD600 nm and confirmed by quantitative culture). Before infection, mice were anesthetized by intraperitoneal injection of a ketamine–xylazine–PBS solution at a dose of 100 mg ketamine/kg of body weight and 10 mg/kg of xylazine. Then mice were infected i.n. with 30 μl of a bacterial suspension containing 2 × 10^5^ CFUs of *S. pneumoniae* or 5 × 10^6^ CFUs of *L. pneumophila*. At the indicated time points after infection, mice were sacrificed for analysis. To determine bacterial burdens, BAL fluid was diluted with distilled H_2_O and spread onto BCYE plates (for *L. pneumophila*) or sheep blood agar plates (for *S. pneumoniae*) and cultured for colony counting.

### Cell Staining and Flow Cytometry

For cell surface staining, single-cell suspensions were incubated with CD16/CD32 FcR blockers (BioLegend) at 4°C for 15 min and followed by staining with fluorescence-conjugated mAbs (mAbs used in this study are listed in **Table S2**) at 4°C for 20 min. After staining, cells were thoroughly washed and resuspended in PBS containing 7-AAD (BioLegend) to exclude dead cells from analysis. For intracellular staining, lung cells were cultured in the presence of Cell Stimulation Cocktail (plus protein transport inhibitors) purchased from eBioscience at 37°C, 5% CO_2_ in a humidified incubator for 3 h. Cells were then stained with surface Abs, fixed, and permeabilized before staining with anti-IL-13 mAb or isotype control (Fixation and Permeabilization Solution Kit, eBioscience). Samples were acquired on Attune NxT cytometer (Thermo Fisher Scientific) and were analyzed with FlowJo software.

### *In Vivo* Cytokine Administration

Recombinant mouse GM-CSF (50 ng, PeproTech), IL-13 (50 ng, PeproTech), CCL2 (6 μg, BioLegend), IL-33 (1 μg, BioLegend), and TGF-β1 (50 ng, PeproTech) were administrated into anesthetized mice *via* i.n. in a volume of 30 μl of PBS every other day for a total of five times. Recombinant CCL2 was used during the first three times of treatment. The treatment begins from day 18 for AM reconstitution analysis or from day 16 for infection experiment after BMT. Control mice were anesthetized and treated with PBS *via* i.n.

### Statistical Analysis

Data are expressed as mean ± SEM. Statistical significance in a two-group comparison was assessed with an unpaired Student’s *t*-test. Correlation between numbers of AMs and neutrophils in BAL fluid and lung tissues after infection was determined by Pearson’s correlation. All statistical analyses were performed with Prism software (GraphPad Software).

## Data Availability Statement

The original contributions presented in the study are included in the article/**Supplementary Material**. Further inquiries can be directed to the corresponding authors.

## Ethics Statement

The animal study was reviewed and approved by Institutional Laboratory Animal Care and Use Committee of Soochow University.

## Author Contributions

CH conceived the study, performed the experiments, analyzed the data, interpreted the results, and wrote the manuscript. HL, RJ, XH, and MC performed the experiments. XD assisted in data analysis and generated the schematic interpretation. FG, HD, and HW assisted in the experimental design and critically discussed the results. X-MG critically reviewed and wrote the manuscript and secured the funding. All authors contributed to the article and approved the submitted version.

## Funding

This work was supported by grant from the National Key Research and Development Program of China (2017YFA0104502) and Priority Academic Program Development of Jiangsu Higher Education Institutions (PAPD).

## Conflict of Interest

The authors declare that the research was conducted in the absence of any commercial or financial relationships that could be construed as a potential conflict of interest.

## Publisher’s Note

All claims expressed in this article are solely those of the authors and do not necessarily represent those of their affiliated organizations, or those of the publisher, the editors and the reviewers. Any product that may be evaluated in this article, or claim that may be made by its manufacturer, is not guaranteed or endorsed by the publisher.

## Supplementary Material

Supplementary Figure 1 Gating strategy for immune cells in BAL fluid and lung tissue. **(A, B)** Debris and doublets were excluded based on FSC and SSC. Dead cells were gated out by 7-AAD staining. Immune cells of lung tissue and BAL fluid in live CD45^+^ cells were analyzed based on cell surface marker expression. CD11c^+^ cells were firstly gated out, when analyzing NK cells, CD19^+^ B cells, CD4^+^ T cells and CD8^+^ T cells, to exclude the interference of high-autofluorescence of AMs.

Supplementary Figure 2 Irradiation abolishes the ability of AMs to proliferate *in vitro*. BAL AMs from lethally irradiated mice or WT mice were cultured *in vitro* in the presence of 30 ng/ml GM-CSF for 6 days. Cell growth on cell culture plates was shown.

Supplementary Figure 3 Normal reconstitution of lung basophils after TCD-BMT. **(A)** Representative plots and **(B)** absolute number of lung basophils (CD11b^+^CD11c^-^FceRIα^+^CD117^-^) in lungs of 28-day allogeneic recipient mice are shown. Data shown are the pooled results of two independent experiments with 3–5 mice per group in each experiment. Data are represented as mean ± SEM.

Supplementary Figure 4 AMs from GVHD mice express high levels of cell surface MHC-I after respiratory bacterial infection. **(A)** 10 days after BMT, allogeneic recipient mice were *i.n.* infected with 5×10^6^ *L. pneumophila.* 48 hrs after infection, BAL fluid cells were harvested and cell surface expression of MHC-I on AMs was analyzed by flow cytometry. **(B)** A statistical analysis of MFI of MHC-I on AMs. Data shown are representative of two independent experiments with 3 mice in each group.

Supplementary Table 1 Sequences of oligonucleotide primers for Quantitative real-time PCR of mouse genes.

Supplementary Table 2 Antibodies used for flow cytometry.
