# Supplementary figures and images for: Cytokine Cocktail Promotes Alveolar Macrophage Reconstitution and Functional Maturation in a Murine Model of Haploidentical Bone Marrow Transplantation
Source: Front Immunol. 2021 Sep 21;12:719727. doi: 10.3389/fimmu.2021.719727 (PMC8490745; doi:10.3389/fimmu.2021.719727)

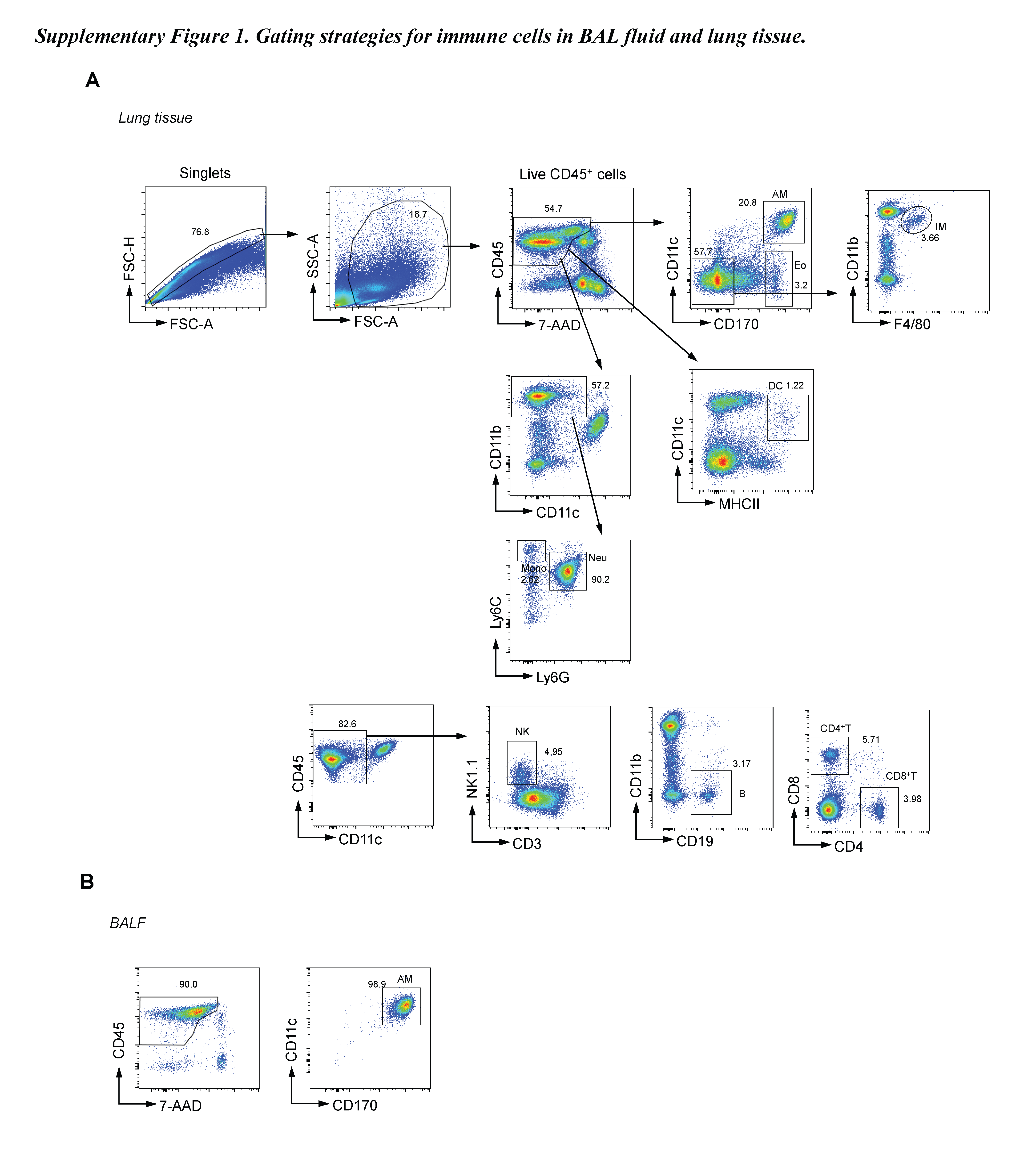

Supplement: Supplementary Figure 1 — Gating strategy for immune cells in BAL fluid and lung tissue. (A, B) Debris and doublets were excluded based on FSC and SSC. Dead cells were gated out by 7-AAD staining. Immune cells of lung tissue and BAL fluid in live CD45+ cells were analyzed based on cell surface marker expression. CD11c+ cells were firstly gated out, when analyzing NK cells, CD19+ B cells, CD4+ T cells and CD8+ T cells, to exclude the interference of high-autofluorescence of AMs. [file Image_1.tif]

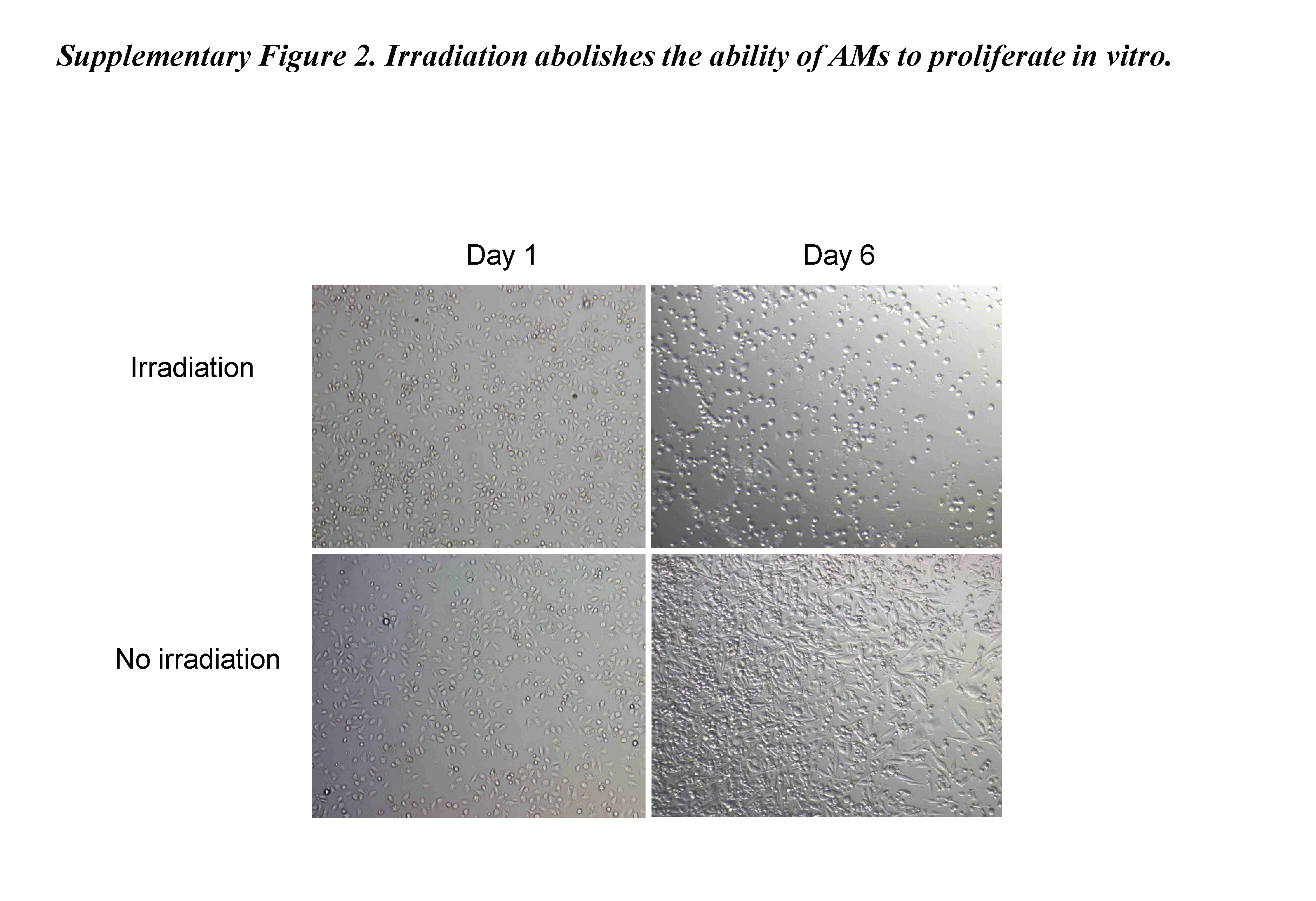

Supplement: Supplementary Figure 2 — Irradiation abolishes the ability of AMs to proliferate in vitro. BAL AMs from lethally irradiated mice or WT mice were cultured in vitro in the presence of 30 ng/ml GM-CSF for 6 days. Cell growth on cell culture plates was shown. [file Image_2.tif]

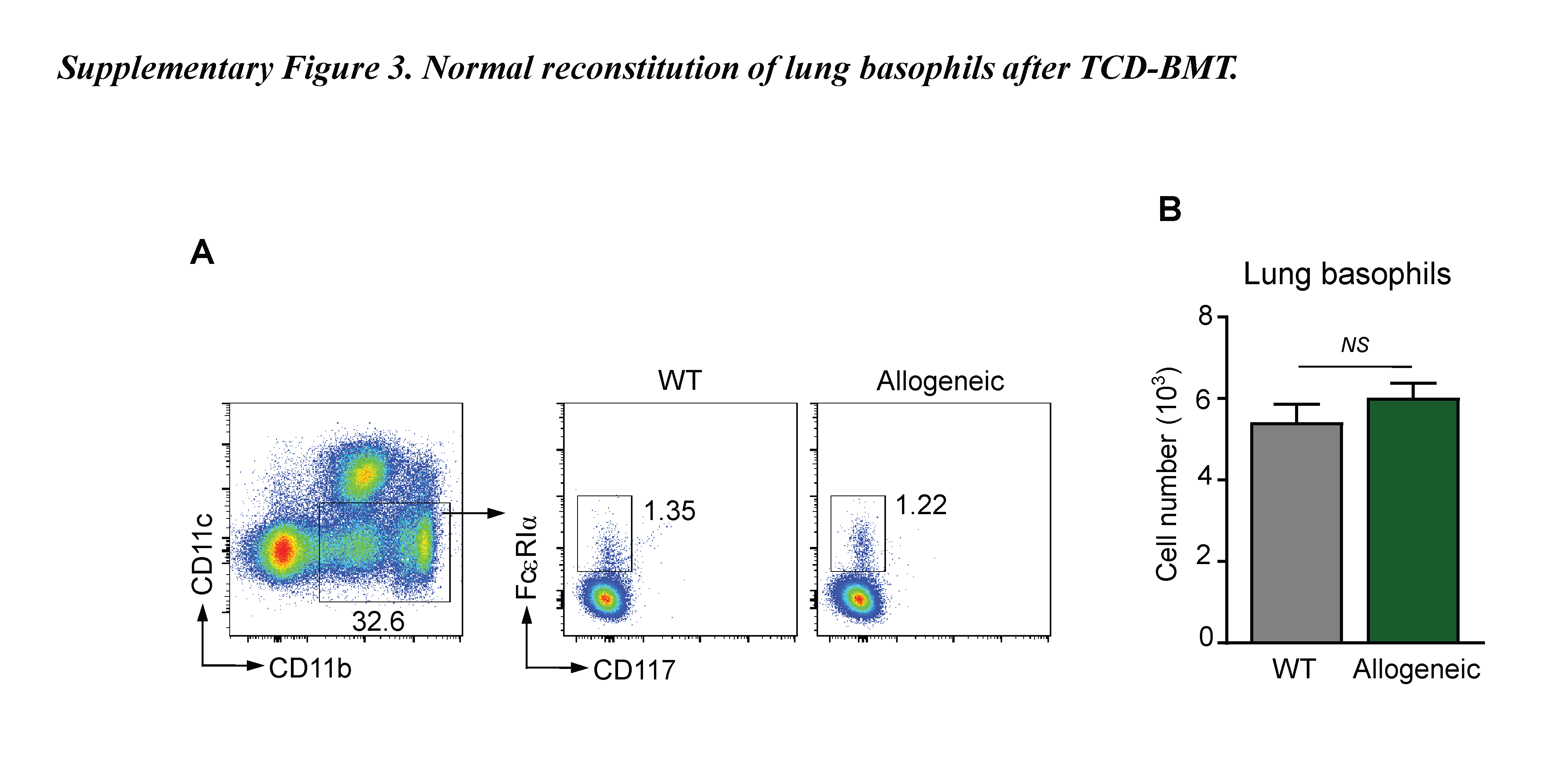

Supplement: Supplementary Figure 3 — Normal reconstitution of lung basophils after TCD-BMT. (A) Representative plots and (B) absolute number of lung basophils (CD11b+CD11c-FceRIα+CD117-) in lungs of 28-day allogeneic recipient mice are shown. Data shown are the pooled results of two independent experiments with 3–5 mice per group in each experiment. Data are represented as mean ± SEM. [file Image_3.tif]

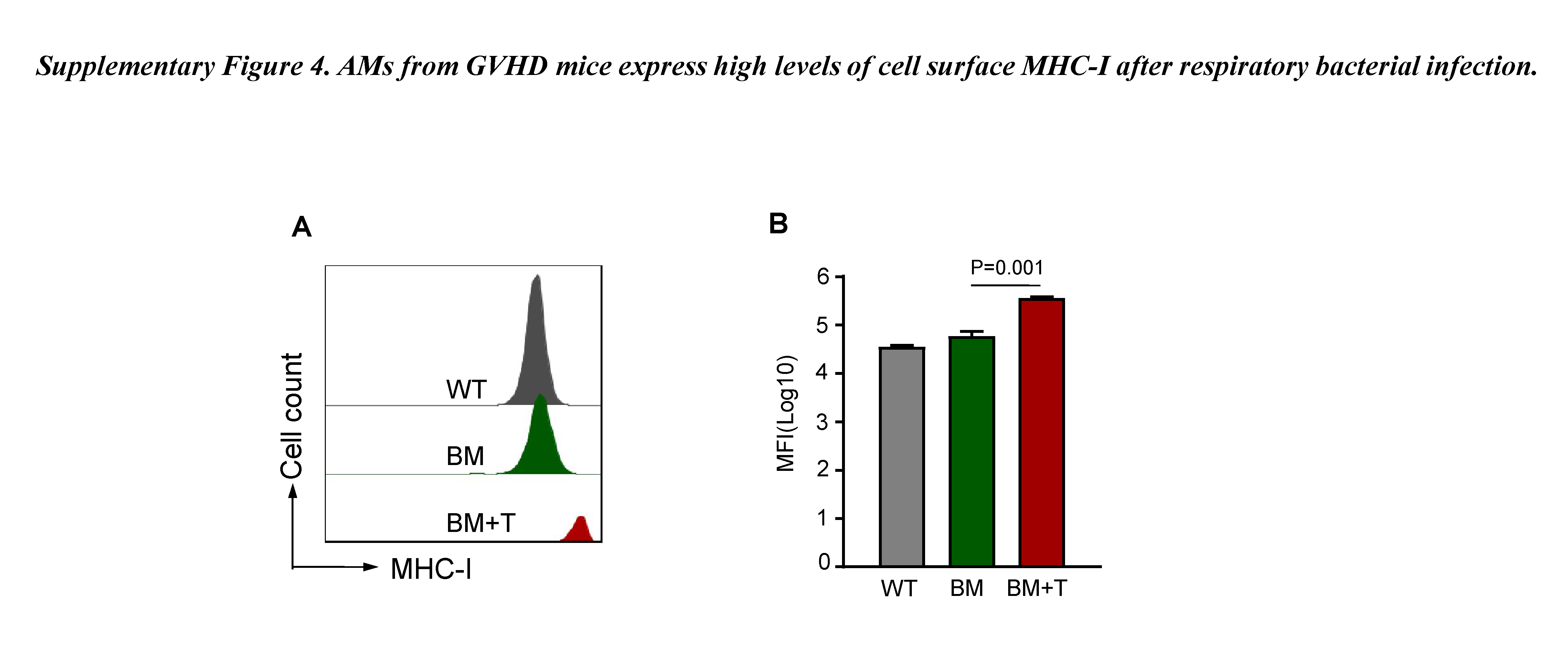

Supplement: Supplementary Figure 4 — AMs from GVHD mice express high levels of cell surface MHC-I after respiratory bacterial infection. (A) 10 days after BMT, allogeneic recipient mice were i.n. infected with 5×10^6^ L. pneumophila. 48 hrs after infection, BAL fluid cells were harvested and cell surface expression of MHC-I on AMs was analyzed by flow cytometry. (B) A statistical analysis of MFI of MHC-I on AMs. Data shown are representative of two independent experiments with 3 mice in each group. [file Image_4.tif]
