# Supplementary material for: Cytokine Cocktail Promotes Alveolar Macrophage Reconstitution and Functional Maturation in a Murine Model of Haploidentical Bone Marrow Transplantation
Source: Front Immunol. 2021 Sep 21;12:719727. doi: 10.3389/fimmu.2021.719727 (PMC8490745; doi:10.3389/fimmu.2021.719727)
Supplement: Supplementary Table 1 — Sequences of oligonucleotide primers for Quantitative real-time PCR of mouse genes. [file Table_1.docx]

**Table S1:** *Sequences of oligonucleotide primers for Quantitative real-time PCR of mouse genes.*

| Primer | Sequence (5’ to 3’) |
| --- | --- |
| *Pparg* Forward | GTGATGGAAGACCACTCGCATT |
| *Pparg* Reverse | CCATGAGGGAGTTAGAAGGTTC |
| *Arg* Forward | CAGAAGAATGGAAGAGTCAG |
| *Arg* Reverse | CAGATATGCAGGGAGTCACC |
| *Ym-1* Forward | TCTGGGTACAAGATCCCTGAACTG |
| *Ym-1* Reverse | GCTGCTCCATGGTCCTTCCA |
| *Mrc-1* Forward | TCTGGGCCATGAGGCTTCTC |
| *Mrc-1* Reverse | CACGCAGCGCTTGTGATCTT |
| *Fizz-1* Forward | TCCAGCTGATGGTCCCAGTG |
| *Fizz-1* Reverse | AAATCCACAAGCACACCCAGT |
| *Clec7a* Forward | GACTTCAGCACTCAAGACATCC |
| *Clec7a* Reverse | TTGTGTCGCCAAAATGCTAGG |
| *Cd11c* Forward | CTGGATAGCCTTTCTTCTGCTG |
| *Cd11c* Reverse | GCACACTGTGTCCGAACTC |
| *Cd206* Forward | CAGGTGTGGGCTCAGGTAGT |
| *Cd206* Reverse | TGTGGTGAGCTGAAAGGTGA |
| *Siglec F* Forward | CCACAGGACCACCCTCTCCTC |
| *Siglec F* Reverse | GGACTTTAGTTCCTGTGTCATCTCCC |
| *Il-13* Forward | CCTGGCTCTTGCTTGCCTT |
| *Il-13* Reverse | GGTCTTGTGTGATGTTGCTCA |
| *Csf-2* Forward | ACATGACAGCCAGCTACTAC |
| *Csf-2* Reverse | TCAAAGGGGATATCAGTCAG |
| *Csf-1* Forward | AGTATTGCCAAGGAGGTGTCAG |
| *Csf-1* Reverse | ATCTGGCATGAAGTCTCCATTT |
| *Il-4* Forward | CGGCATTTTGAACGAGGTCACAGG |
| *Il-4* Reverse | AGCACCTTGGAAGCCCTACAGACG |
| *Il-33* Forward | TGAGACTCCGTTCTGGCCTC |
| *Il-33* Reverse | CTCTTCATGCTTGGTACCCGAT |
| *Tgf-b1* Forward | TGACGTCACTGGAGTTGTACGG |
| *Tgf-b1* Reverse | GGTTCATGTCATGGATGGTGC |
| *Areg* Forward | GGTCTTAGGCTCAGGCCATTA |
| *Areg* Reverse | CGCTTATGGTGGAAACCTCTC |
| *Gapdh* Forward | CTGCCCAGAACATCATCCCT |
| *Gapdh* Reverse | ACTTGGCAGGTTTCTCCAGG |
| *Hprt* Forward | GTTAAGCAGTACAGCCCCAAAATG |
| *Hprt* Reverse | AAATCCAACAAAGTCTGGCCTGTA |

**Table S2:** *Antibodies used for flow cytometry.*

| Antibody | Manufacturer | ID |
| --- | --- | --- |
| AF700 anti-mouse CD45 (30-F11) | eBiosciences | Cat# 56-0451-82 |
| APC-Cy7 anti-mouse CD11c (N418) | Biolegend | Cat# 117323 |
| APC anti-mouse CD19 (6D5) | Biolegend | Cat#115512 |
| FITC anti-mouse CD11b (M1/70) | Biolegend | Cat#101206 |
| PE anti-mouse CD4 (GK1.5) | Biolegend | Cat#100407 |
| PE-Cy7 anti-mouse CD8 (53-6.7) | Biolegend | Cat#100721 |
| PE-CF594 anti-mouse NK1.1 (PK136) | BD Biosciences | Cat#562864 |
| Pacific blue anti-mouse CD3e (145-2C11) | Biolegend | Cat#100333 |
| BV501 anti-mouse Ly6C (HK1.4) | Biolegend | Cat#128033 |
| APC-Cy7 anti-mouse I-A/I-E (M5/114.15.2) | Biolegend | Cat#107627 |
| APC anti-mouse F4/80 (BM8) | Biolegend | Cat#123115 |
| PE anti-mouse Siglec F (1RNM44N) | eBiosciences | Cat#12-1702-80 |
| PE-Cy7 anti-mouse Ly6G (1A8) | Biolegend | Cat#127617 |
| PE-Dazzle 594 anti-mouse CD11c (N418) | Biolegend | Cat#117347 |
| Pacific blue anti-mouse Ly6G(1A8) | Biolegend | Cat#127611 |
| APC-Cy7 anti-mouse CD117 (ACK2) | Biolegend | Cat#135135 |
| AF647 anti-mouse F4/80 (BM8) | Biolegend | Cat#123121 |
| PE-Cy7 anti-mouse FcεRIα (MAR-1) | Biolegend | Cat#134318 |
| APC anti-mouse CD90.2(30-H12) | Biolegend | Cat#105311 |
| FITC-Lineage cocktail | Biolegend | Cat#133301 |
| Percp-Cy5.5 anti-mouse CD25(PC61) | Biolegend | Cat#102029 |
| PE-Cy7 anti-mouse CD127 (A7R34) | Biolegend | Cat#135013 |
| PE anti-mouse ST2(DIH9) | Biolegend | Cat#145304 |
| AF647 anti-mouse H-2D^d^ (34-2-12) | Biolegend | Cat#110612 |
| PE anti-mouse LPAM-1(DATK32) | Biolegend | Cat#120605 |
| APC anti-mouse TER-119 (TER-119) | Biolegend | Cat#116212 |
| APC anti-mouse CD135 (A2F10) | Biolegend | Cat#135309 |
